# Carbon Nanotubes and Nanosheets for Sustainable Solutions

**DOI:** 10.3390/nano14201649

**Published:** 2024-10-14

**Authors:** Muralidharan Paramsothy

**Affiliations:** NanoWorld Innovations (NWI), 1 Jalan Mawar, Singapore 368931, Singapore; mpsothy@yahoo.co.uk

This Special Issue is a continuation of the preceding Special Issue titled “70th Year Anniversary of Carbon Nanotube Discovery—Focus on Real World Solutions”. As previously mentioned, 70 years ago, in 1952, Russian scientists L.V. Radushkevich and V.M. Lukyanovich published clear images showing multiwalled carbon nanotubes (MWCNTs) having 50 nm diameter [1]. Their paper was in Russian, published in the then Zhurnal Fizicheskoi Khimii (now Russian Journal of Physical Chemistry A), at the height of the Cold War. It is noteworthy that S. Iijima’s work, published some 30 years ago in 1991, also generated unprecedented interest in carbon nanostructures, including CNTs, and has since fueled intense research in the area of nanotechnology [2]. *Radushkevich and Lukyanovich should be credited for the discovery that carbon filaments could be hollow and have a nanometer-size diameter, that is to say for the discovery of CNTs* [3]. 

CNTs are recognized for ultrahigh strength and deformability, high thermal conductivity, ballistic electrical conductivity, selected biocompatibility, unusual optical properties, and high surface area. Graphene and nanohorns are other well-known nanoscale forms of carbon, but CNTs remain distinguished by virtue of their one-dimensional or 1D structure, which enables the directional tailoring of exceptionally favorable characteristics (enabling the desired anisotropic properties) when required in application. Towards the contemporary theme of sustainability, twelve representative articles, as listed below, have been published in this Special Issue. The first eight articles are on currently impactful applications utilizing CNTs and/or graphene, while the last four articles are on synthesis and nanoscale fundamental aspects of CNTs and/or graphene.

Geographically, including mixed country authorship, "this Special Issue continuation is also of a truly global nature: 5 articles are from the Republic of Korea, 4 from Italy, 3 from USA, and one from each of the following countries: Pakistan, Malaysia, China, Japan, Taiwan, Bulgaria, Hungary, Germany, The Netherlands, and Belgium. 

## 1. Biomedical Application: Seratonin Nanosensors

*Innovatively combining directed evolution and machine learning-based prediction*, An and Jeong et al. (in Contribution 1) employed a novel approach to improve serotonin-responsive ssDNA-wrapped single-walled carbon nanotube (ssDNA-SWCNT) nanosensors. An optimization process targeting the sensitivity and selectivity of ssDNA-SWCNT nanosensors was used. In the three rounds for higher serotonin sensitivity, sensitivity was substantially improved. A remarkable 2.5-fold enhancement in fluorescence response compared to the original sequence was achieved. Next, there were two rounds of focusing on selectivity for serotonin over dopamine. Despite the structural similarity between the two neurotransmitters, a 1.6-fold increase in selectivity was achieved. *This methodology offered high-throughput screening of mutated sequences, marking a significant advancement in biosensor development. The top-performing nanosensors, N2-1 for sensitivity and L1-14 for selectivity, present promising reference sequences for future studies involving serotonin detection.*

## 2. Agriculture Application: Seed Priming

*In creatively and synergistically working with nature*, Krumova and Velikova et al. (in Contribution 2) explored the effect of seed priming with single-walled carbon nanotubes grafted with Pluronic P85 polymer (P85-SWCNT) on Pisum sativum (var. RAN-1) seed germination, as well as early stages of plant development, leaf anatomy, and photosynthetic efficiency. The effects in relation to control and P85-primed seeds were observed and evaluated. The data clearly revealed that seed priming with P85-SWCNT was safe for the plant since it did not impair the seed germination, plant development, leaf anatomy, biomass, or photosynthetic activity and even increased the amount of photochemically active photosystem II centers in a concentration-dependent manner. Only 300 mg/L concentration exerted an adverse effect on those parameters. The P85 polymer, however, was found to exhibit a number of negative effects on plant growth (i.e., root length, leaf anatomy, biomass accumulation, and photoprotection capability), most probably related to the unfavorable interaction of P85 unimers with plant membranes. *The findings substantiated the future exploration and exploitation of P85-SWCNT as nanocarriers of specific substances promoting not only plant growth at optimal conditions but also better plant performance under a variety of environmental stresses. The evaluation of P85-SWCNT toxicity also required further dedicated study.*

## 3. Energy Application: Superconductivity

*In a critical approach*, Abdullah and Kechik et al. (in Contribution 3) systematically investigated the effects of graphene addition on the phase formation and superconducting properties of (Bi_1.6_Pb_0.4_)Sr_2_Ca_2_Cu_3_O_10_ (Bi-2223) ceramics synthesized using the co-precipitation method. Samples of Bi-2223 with different weight percentages of graphene nanoparticles were studied. The sample phase formations and crystal structures were characterized via X-ray diffraction (XRD), while the superconducting critical temperature (T_c_) was investigated using alternating current susceptibility (ACS). The XRD showed a larger proportion of high-T_c_ phase (Bi-2223) co-existing with a smaller portion of low-T_c_ phase (Bi-2212) in the samples prepared. The volume fraction of the Bi-2223 phase increased for the samples with x = 0.3 wt.% and 0.5 wt.% of graphene and slightly decreased at x = 1.0 wt.% graphene concentration. The ACS showed that the onset critical temperature (T_c-onset_), phase lock-in temperature (T_cj_), and coupling peak temperature (T_P_) decreased when graphene was added to the samples. *Adding graphene nanoparticles to Bi-2223 samples improved the pinning center and hence enhanced the critical current density (Jc) of Bi-2223 superconductors, paving the way for impactful future study and application.*

## 4. Energy Application: Supercapacitor

*Using eco-friendly, low-cost materials and a simple technology*, Arena and Scandurra et al. (in Contribution 4A) developed solid-state supercapacitors on paper substrates with areal capacitance in the order of 100 mF⋅cm^−2^. The electrochemically active material used as the electrode was prepared from a stable water-based ink obtained by doping commercial polypyrrole (PPY) powder with dodecylbenzene sulfonic acid (DBSA) and characterized by optical and electrical measurements, Raman investigation, and atomic force microscopy. The PPY:DBSA ink was directly applied to paper by means of rechargeable water pens, obtaining electrically conducting solid state tracks after drying. The PPY:DBSA layers were then interfaced with one another using a polymer gel based on potassium hydroxide and chitosan, acting both as the ion-conducting medium as well as the separator. *The areal capacitance of the devices developed with this simple method was improved when nanostructured carbon material without any toxic, rare, or precious chemical element or component was incorporated into the PPY:DBSA ink.*

*Towards environmental sustainability*, Choi and Jeon et al. (in Contribution 4B) explored the electrochemical wetting method in depth as an effective strategy for functionalization of carbon nanotube (CNT) sheets. The electrochemical wetting method is known to offer a range of advantages over other functionalization techniques, such as plasma treatment and acid wetting. While plasma treatment necessitates specialized equipment and can be both energy-intensive and time-consuming, acid wetting often involves the use of hazardous chemicals and stringent safety protocols. Electrochemical wetting, on the other hand, is characterized by its simplicity, cost-effectiveness, and efficiency; it employs a straightforward electrochemical setup and does not require specialized gasses or chemicals. It was shown that electrochemical wetting induced a significant transition from hydrophobic to hydrophilic behaviors, as evidenced by the dramatic decrease in the contact angles of water droplets on the CNT surfaces. The introduction of oxygen-containing functional groups and electrolyte-infiltration-induced inter-nanotube swelling were identified as the key mechanisms behind this transition. *Moreover, this study demonstrated a remarkable improvement in the supercapacitor performance of the electrochemically wetted CNT sheet. The environmental sustainability of the electrochemical wetting method is an important consideration, especially in the current global context of growing environmental concerns. While the electrochemical wetting process is relatively straightforward and does not require hazardous chemicals, a thorough environmental impact assessment is still essential.*

*Groundbreakingly,* Choi et al. (in Contribution 4C) introduced a novel approach to addressing the low electrical double-layer capacitance of carbon nanotube (CNT) sheets in the development of flexible, high-performance supercapacitors as a focal point in energy storage research. Electrochemical oxidation treatment of CNT sheets stacked on a polyethylene terephthalate substrate was performed to introduce oxygen-containing functional groups onto the CNT surface. This dramatically enhanced the pseudocapacitive effect and improved ion adsorption characteristics. Consequently, using the material in a two-electrode system increased the capacitance by 54 times compared to pristine CNT. The results of electrochemical performance characterization, including cyclic voltammograms, galvanostatic charge/discharge curves, and capacitance retention testing data, confirmed the efficacy of the electrochemical oxidation approach. Furthermore, the mechanical flexibility of the electrochemically wetted CNT sheets was validated through resistance and discharge retention testing under repetitive bending (98% capacitance retention after 1000 bending cycles). *The results demonstrated that electrochemically wetted CNT sheets retained their intrinsic mechanical and electrical properties while significantly enhancing the electrochemical performance (0.59 mF/cm^2^ or 97.8 F/g). This work represents a significant advancement in the development of flexible, high-performance supercapacitors with potential applicability to wearable electronics, flexible displays, and next-generation energy storage solutions.*

## 5. Thermal Application: Nanocomposite for Road De-Icing

*In a most practical way that impacts everyday life*, Jang and Cho et al. (in Contribution 5) examined the relationship between the concentration of carbon nanotubes (CNTs) and supply voltage with respect to the heating and electrical performance of CNT/epoxy (EP) coating and assessed the impact of atmospheric temperature on this relationship. The goal was to provide an alternative solution to the issues caused by chemical de-icing agents in road systems. By dispersing CNTs in an epoxy matrix, highly electrically conductive coatings were successfully fabricated. Measurement of the variation of electrical conductivity with CNT concentration showed that the percolation threshold occurred when CNT concentration was between 0.5 wt.% and 1.0 wt.%. To evaluate the applicability of CNT/EP coatings based on Joule heating, thermal efficiency was investigated at room temperature, as it signified the de-icing capability in relation to the energy consumed. Results showed that the CNT/EP coating had high thermal efficiency by attaining maximum temperatures with relatively low electric power. As a result of the heating test of the CNT/EP coating according to atmospheric temperature, it was found that a decrease in atmospheric temperature reduced the heating performance of the coating. Nevertheless, it was confirmed that the heating performance of the coating remained above freezing even under sub-zero temperatures. Finally, application tests confirmed that the de-icing coating developed and verified in this study presented good heating performance and applicability. *As a result of the application test at sub-zero temperature, it was proved that the coating could reach the de-icing temperature, and it was possible to respond according to the installation environment. Through these results, it was confirmed that the developed coating could be used not only to prevent ice on the road system as an eco-friendly coating but also be applied to structures in various fields.*

## 6. Structural Application: Vibration and Damping

*Very insightfully at the nanoscale*, Strozzi and Elishakoff et al. (in Contribution 6) conducted a comparison between three classical shell theories as applied to the linear vibrations of single-walled carbon nanotubes (SWCNTs); specifically, the evaluation of the natural frequencies was conducted via Donnell, Sanders, and Flügge shell theories. The actual discrete SWCNT was modeled by means of a continuous homogeneous cylindrical shell while considering equivalent thickness and surface density. Taking into account the intrinsic chirality of CNTs, a molecular based anisotropic elastic shell model was considered. Simply supported boundary conditions were imposed, and a complex method was applied to solve the equations of motion and obtain the natural frequencies. Comparisons with the results of molecular dynamics simulations available in the literature were performed to check the accuracy of the three different shell theories, where the Flügge shell theory was found to be the most accurate. A parametric analysis evaluating the effect of diameter, aspect ratio, and number of waves along the longitudinal and circumferential directions on the natural frequencies of SWCNTs was then performed in the framework of the three different shell theories. Assuming the results of the Flügge shell theory as a reference, it was obtained that the Donnell shell theory was not accurate for relatively low longitudinal and circumferential wavenumbers, for relatively low diameters, and for relatively high aspect ratios. *On the other hand, it was found that the Sanders shell theory was very accurate for all the considered geometries and wavenumbers, and therefore it could be correctly adopted instead of the more complex (and computationally expensive) Flügge shell theory for the vibration modeling of SWCNTs.*

## 7. Synthesis and Structure: Nanofiber Growth on Nanowire

*Pioneers at the nanoscale*, Rudolf and Cepek et al. (in Contribution 7) successfully synthesized carbon nanofiber (CNF)-InAs hybrid nanostructures using chemical vapor deposition (CVD) at optimized growth conditions. The original ensemble of InAs nanowires was thermally stable and preserved during annealing, hydrogen pre-treatment, and CVD growth of CNFs. When the growth was performed only with acetylene, carbon nanoparticles (C NPs) were found on the surface and on the InAs nanowires (NWs); C NPs were absent when hydrogen was added. In the latter condition, Raman spectroscopy revealed the presence of graphitic-like carbon structures with a high number of defects, which pointed to CNF formation. The CNFs preferentially nucleated at the tip of the InAs NWs, probably catalyzed by a gold–indium (Au-In) alloy. The number density of CNFs of decorated InAs NWs increased from 15% to 50% when Fe was added as a catalyst in the CVD process. *The results demonstrated that interconnections between adjacent InAs NWs with carbon fibers could be obtained via CVD. However, connections made between neighboring NWs were unsystematic. These results represent a compelling example towards controlled interconnections between adjacent InAs NWs formed by CNFs. The integration of carbon nanostructures with semiconductor NWs holds significant potential for energy-efficient integrated circuits.*

## 8. Electronic Structure: Pseudopotential Method

*Concerning nanoscale electronic behavior*, Chang et al. (in Contribution 8) implemented a semi-empirical pseudopotential (SEP) method for calculating the band structures of graphene and graphene nanoribbons. The basis functions adopted were two-dimensional plane waves multiplied by several B-spline functions along the perpendicular direction. The SEP included both local and non-local terms, which were parametrized to fit relevant quantities obtained from the first-principles calculations based on the density-functional theory (DFT). With only a handful of parameters, the full band structure of graphene obtained by DFT was reproduced with a negligible difference. The method was simple to use and more efficient than the DFT calculation. The SEP method was applied to calculate the band structures of graphene nanoribbons. By adding a simple correction term to the local pseudopotentials on the edges of the nanoribbon (which mimics the effect caused by edge creation), band structures of the armchair nanoribbon that were fairly close to the results obtained by DFT were obtained again. *This approach allows the simulation of the optical and transport properties of realistic nanodevices made of graphene nanoribbons with very little computation effort.*

## 9. Crystallographic Structure: Defect Study

*Fundamentally for nanoscale functionalization,* Tayyab et al. (in Contribution 9) investigated highly aligned multi-wall carbon nanotubes (MWCNTs) using scanning electron microscopy (SEM), Raman spectroscopy, and X-ray photoelectron spectroscopy (XPS) before and after bombardment performed using noble gas ions of different masses (argon, neon, and helium) in an ultra-high-vacuum (UHV) environment. Ion irradiation led to changes in morphology, deformation of the carbon (C) honeycomb lattice, and different structural defects in MWCNTs. One of the major effects was the production of bond distortions, as determined by micro-Raman and micro-XPS. An increase in sp^3^ distorted bonds at higher binding energy with respect to the expected sp^2^ associated signal of the carbon 1s core level, as well as an increase in dangling bonds, was observed. Furthermore, the surface damage as determined by the XPS carbon 1s core level was equivalent upon bombarding with ions of different masses, while the impact and density of defects in the lattice of the MWCNTs as determined by micro-Raman were dependent on the bombarding ion mass; heavier for helium ions and lighter for argon ions. *These results on the controlled increase in sp^3^ distorted bonds, as created on the MWCNTs, open new functionalization prospects to improve and increase atomic hydrogen uptake on ion-bombarded MWCNTs.*

## 10. Crystallographic Structure: Defect Engineering

*Futuristically for 2D nanomaterials,* Hofsäss and Junge et al. (in Contribution 10) showed that by using electrostatic masks and potential differences, graphene could be doped with different fluences in only one implantation step and that by adjusting the deflection voltage, the transition region between the doped and un-doped regions could be set. This is a great step towards the production of electronic graphene devices such as transistors using ion implantation, as by being able to implant a sample at different energies at different surface positions, future measurements and investigations can be accelerated and therefore eliminate unintended experimental variations in sample preparation as only one sample needs to be implanted instead of multiple. Further experiments to sharpen the transition zone are planned, as the simulations carried out can confirm the findings from the experiments and are therefore a valuable tool for predicting the processes during ultra-low energy ion implantation of graphene (2D) materials. *These methods can be transferred to other 2D materials such as transition-metal dichalcogenides (TMDs) and therefore can be helpful for a better investigation and manipulation of these materials.*
